# Gas therapy: an innovative application for intervertebral disc degeneration

**DOI:** 10.4103/mgr.MEDGASRES-D-25-00191

**Published:** 2026-01-06

**Authors:** Lu Cai, Bin Ru, Haijiang Ren, Fang Cai, Lingyuan Zeng, Jiayu Yang, Shibo Wang, Han Zhang, Yao Li, Long Zhang

**Affiliations:** 1 Department of Painology, Shaoxing Second Hospital, Shaoxing, Zhejiang Province, China; 2 Center for Rehabilitation Medicine, Department of Pain Management, Zhejiang Provincial People’s Hospital, Affiliated People’s Hospital, Hangzhou Medical College, Hangzhou, Zhejiang Province, China; 3 Department of Orthopedics, The First Affiliated Hospital, Zhejiang University School of Medicine, Hangzhou, Zhejiang Province, China; 4 Department of Orthopedics, The Second Hospital of Shanxi Medical University, Shanxi Medical University, Taiyuan, Shanxi Province, China; 5 Institute for Smart Biomedical Materials, School of Materials Science & Engineering, Zhejiang Sci-Tech University, Hangzhou, Zhejiang Province, China

**Keywords:** gas, gas therapy, hydrogen, hydrogen sulfide, hyperbaric oxygen, intervertebral disc degeneration, low back pain, nitric oxide, nucleus pulposus, ozone

## Abstract

**Facts**

1. Gas therapy represents a novel and promising therapeutic paradigm for intervertebral disc degeneration, utilizing bioactive gases to modulate oxidative stress, inflammation, and extracellular matrix metabolism.
2. Certain gas therapies, such as medical ozone and hyperbaric oxygen, have already been translated into clinical use for intervertebral disc degeneration, demonstrating efficacy in pain alleviation, disinfection, and improving functional outcomes through minimally invasive delivery.
3. The core mechanisms of gas therapeutics involve the restoration of disc microenvironment homeostasis via specific actions, including reactive oxygen species scavenging, suppression of inflammatory cytokines, inhibition of inflammasome activity, and enhancement of collagen synthesis.
4. Combination strategies integrating gas therapy with other regenerative approaches—such as stem cell transplantation, bioactive scaffolds, or drug delivery systems—exhibit synergistic potential for amplifying anti-inflammatory, antioxidant, and anabolic effects in disc repair.

**Open questions**

1. What are the precise molecular mechanisms and signaling pathways (e.g., hydrogen-mediated nuclear factor erythroid 2-related factor 2 activation, hydrogen sulfide-dependent extracellular matrix regulation) through which gaseous mediators exert their therapeutic effects in human disc cells under pathological microenvironments?
2. How can physiologically relevant disease models—such as human disc organoids or large animal models under biomechanical loading—be developed and utilized to better recapitulate human intervertebral disc degeneration pathophysiology and improve the translational validity of preclinical gas therapy research?
3. What is the clinical efficacy and safety of gas therapeutic protocols in large-scale, multicenter randomized controlled trials? How can standardized treatment parameters and personalized regimens be established for different subtypes and etiologies of intervertebral disc degeneration?

Environmental gaseous molecules extensively participate in human physiological and pathological regulation through differential biological effects. Gas transmitter-based therapeutic strategies, as emerging intervention modalities, have demonstrated significant translational value in intervertebral disc degeneration management. The intervertebral disc degeneration susceptibility to progressive degenerative pathology stems from its unique avascular nature and complex biomechanical microenvironment, while conventional therapies face limitations in efficacy and carry invasive risks. This review systematically delineates innovative applications of gaseous therapeutics for intervertebral disc degeneration, encompassing clinically established ozone and hyperbaric oxygen therapies alongside preclinical-stage hydrogen, hydrogen sulfide, and nitric oxide interventions. Comprehensive analyses address molecular properties, biological functions, and mechanistic actions. Current evidence indicates that gas therapies significantly alleviate pain and improve functional impairment through targeted modulation of oxidative stress–inflammation–apoptosis cascades and extracellular matrix metabolic dysregulation. Their minimally invasive precision delivery capabilities and multimodal bio-regulatory advantages offer groundbreaking diagnostic and therapeutic strategies for intervertebral disc degeneration, exhibiting well-defined clinical translation potential.

## Introduction

Low back pain is a prevalent orthopedic condition frequently resulting in activity limitation.^1^,^2^ Currently, intervertebral disc degeneration (IVDD) is widely recognized as its primary etiology; however, the underlying molecular mechanisms remain elusive.^3^ The intervertebral disc (IVD) is a structurally specialized organ composed of the nucleus pulposus (NP), annulus fibrosus, and cartilaginous endplates, which collectively maintain spinal load-bearing capacity and dissipate rotational stresses. As the largest avascular organ in the human body, the IVD relies exclusively on diffusion-mediated nutrient exchange through the capillary plexuses within the cartilaginous endplates. This unique metabolic dependency renders the IVD particularly vulnerable to degenerative cascades. Current therapeutic strategies primarily consist of conservative management and end-stage surgical intervention: conservative approaches offer only temporary symptomatic relief with a high recurrence rate, while surgery, although capable of removing the diseased disc, carries risks such as adjacent segment degeneration, and internal fixation devices inevitably restrict spinal mobility.^4^,^5^,^6^ Consequently, elucidating the molecular pathogenesis of IDD and identifying precise and effective therapeutic targets represent a pivotal approach to preventing and treating IVD degeneration and injury.

As critical endogenous mediators, gas signaling molecules—including nitric oxide (NO), hydrogen sulfide (H_2_S), and hydrogen (H_2_)—facilitate cellular communication and maintain physiological homeostasis. The foundation of gas therapy stems from the landmark discovery of NO as the first endogenous gaseous mediator, recognized by the 1998 Nobel Prize in Physiology or Medicine for its cardiovascular therapeutic implications.^7^ This innovative approach exploits bioactive regulatory functions, achieved through exogenous supplementation or endogenous modulation, to restore physiological balance and combat pathology. Distinct advantages over conventional treatments include superior deep-tissue penetration, multi-target modulation, minimal systemic toxicity facilitated by endogenous metabolic clearance, favorable biosafety, and cost-efficiency. Collectively, these properties overcome therapeutic limitations like drug resistance and surgical constraints, positioning gas therapy as a promising precision medicine strategy. Clinically, these benefits are evidenced in NO inhalation therapy for neonatal respiratory disorders, such as persistent pulmonary hypertension,^8^ and in ozone (O_3_) or hyperbaric oxygen (HBO) therapy for degenerative spinal conditions.^9^,^10^ Preclinical research prioritizes gas therapy for intractable bone pathologies such as IVDD, where H_2_, H_2_S, and NO serve as targeted agents, either replacing or augmenting conventional treatments. Notwithstanding this progress, current IVDD investigations remain restricted to a narrow spectrum of gaseous mediators. Consolidating knowledge on existing gas signaling molecules’ regulatory mechanisms and biological functions is therefore essential. Furthermore, leveraging this foundation to discover novel therapeutic gaseous mediators represents a critical research imperative.

**Figure 1:**
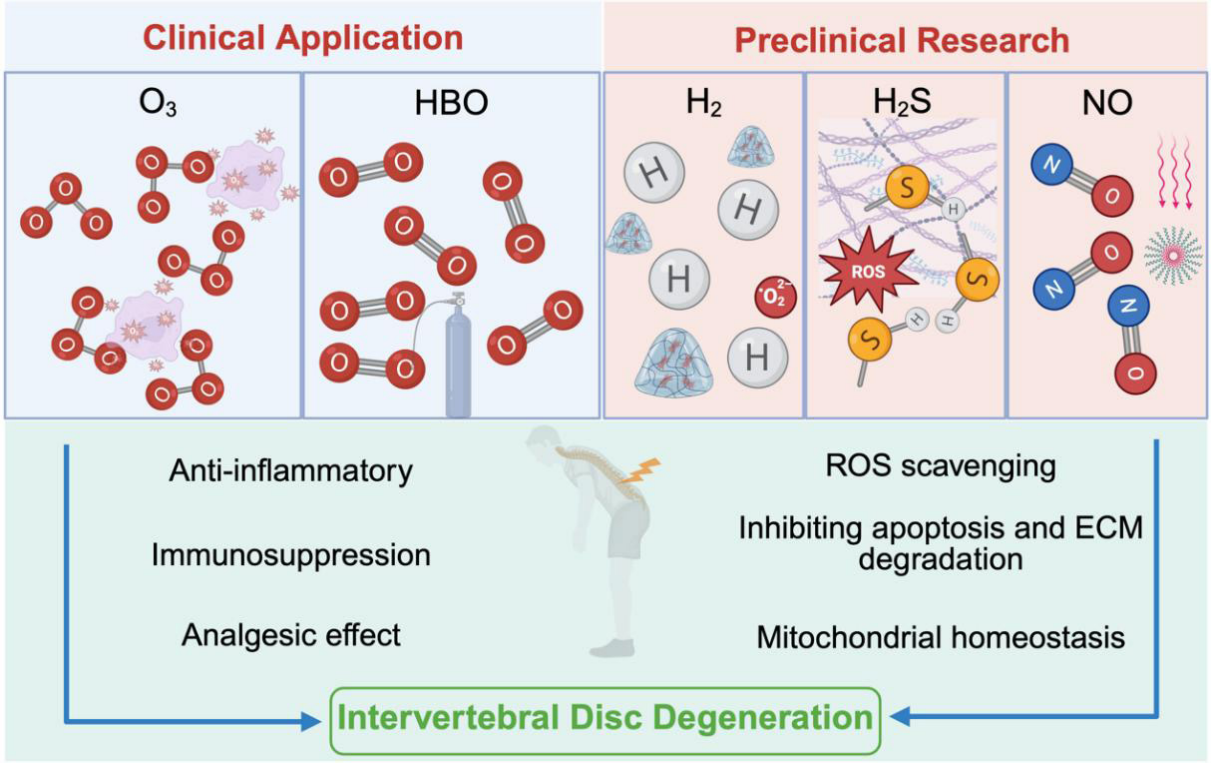
The involvement of various metal or metal oxide NPs in the treatment of intervertebral disc degeneration. This review comprehensively delineates gas therapeutics (O_3_, HBO, H_2_, H_2_S, and NO) for intervertebral disc degeneration, integrating clinical applications with preclinical mechanistic insights. Created with BioRender.com. ECM: Extracellular matrix; H_2_: hydrogen; H_2_S: hydrogen sulfide; HBO: hyperbaric oxygen; NO: nitric oxide; NP: nanoparticle; O_3_: ozone; ROS: reactive oxygen species.

This review comprehensively elucidates recent advances in gaseous mediator applications for IVDD, spanning both clinical practice and preclinical research. We focus on their physiological mechanisms and therapeutic actions, delineating gas therapy advantages at the molecular level while outlining future research trajectories in this field (**Figure 1**).

## Search Strategy

A targeted PubMed search (https://pubmed.ncbi.nlm.nih.gov/) was conducted to identify relevant publications on gaseous mediators in IVDD. The search encompassed key terms including “gas therapy,” “hydrogen sulfide,” “nitric oxide,” “ozone,” “hyperbaric oxygen,” and “intervertebral disc degeneration,” published from 2008 to 2025. The selection focused on original research and authoritative reviews published in English, prioritizing studies elucidating molecular mechanisms or therapeutic applications of gaseous mediators in IVDD. This approach ensured the inclusion of high-quality evidence central to the review’s scope.

## Gases in Intervertebral Disc Degeneration Management: From Bench to Bedside

### Ozone

O_3_, an unstable triatomic oxygen allotrope, exhibits higher aqueous solubility than molecular oxygen (O_2_). Initially employed in medical settings during the early 20^th^ century, O_3_ therapy gained prominence for treating gangrene during World War I.^11^ Post-war applications expanded to include wound disinfection and healing,^12^ as well as management of hepatitis,^13^ and arthritis.^14^ Its favorable safety profile, procedural simplicity, and cost-effectiveness have established O_3_ therapy as a validated complementary or alternative medical modality. O_3_ induces overexpression of antioxidant enzymes (e.g., superoxide dismutase) to scavenge reactive oxygen species (ROS) such as hydrogen peroxide (H_2_O_2_), while modulating inflammatory homeostasis through upregulating anti-inflammatory factors interleukin (IL)-10 and transforming growth factor-β and suppressing pro-inflammatory mediators IL-1β, IL-6 and transforming growth factor-α.^15^ Mechanistically, it mediates endothelial release of NO and platelet-derived growth factor, enhancing microcirculation and alleviating venous stasis, thereby reducing ischemic edema in nerve roots.^16^ O_3_ injection therapy targeting the IVD and peripheral nerve roots has emerged as a well-established intervention for disc herniation. This modality is clinically adopted across many countries, particularly Italy, Canada and China.^17^,^18^

O_3_ intradiscal injection constitutes a minimally invasive therapeutic intervention for discogenic sciatica refractory to conventional conservative management. This approach leverages the biochemical properties of O_3_, primarily involving the oxidation of proteoglycans within the NP, to induce targeted decompression and potential anti-inflammatory effects within the affected IVD. Clinical evidence supports its favorable safety profile and significant efficacy in achieving meaningful pain reduction and functional improvement over short-to-medium-term follow-up periods. The procedure is predominantly performed under digital subtraction angiography guidance using O_3_ concentrations of 25, 30, or 40 μg/mL. Alternatively, computed tomography-guided administration may be utilized in select cases.^19^,^20^,^21^,^22^ Clinical trials demonstrate O_3_ therapy’s superior efficacy compared to laser disc decompression. Ozone nucleolysis effectively relieves pain in herniated disc patients unresponsive to conservative treatment, with or without steroid injection.^23^,^24^ For lumbar disc herniation, O_3_-augmented percutaneous discectomy alleviates neuropathic pain from nerve root compression and inflammatory stimulation while improving motor impairment.^20^ When combined with radiofrequency ablation, although no significant short-term (1 month) advantage over radiofrequency ablation monotherapy was observed, longitudinal data demonstrate superior pain reduction with combination therapy at 1-year follow-up.^25^ Research has elucidated key mechanisms underlying O_3_ therapy for lumbar disc herniation: (1) inhibition of local inflammation,26 (2) oxidizing the proteoglycans contained in NP, dehydration with a reduction of the disc volume and pressure,^27^ and (3) fibroblast proliferation stimulation.^28^ These mechanisms collectively mitigate nerve compression while restoring oxidative homeostasis (**Table 1**).^23^,^24^,^29^,^30^,^31^,^32^,^33^,^34^

**Table 1:** Randomized controlled trials of ozone on intervertebral disc degeneration

Study	Sample size	Inclusion criteria	Outcome measure
Gallucci et al.^29^	77	Monoradicular pain; disk herniation; herniation site congruous with the neurologic level; Oswestry Disability Index > 30%; symptoms > 8 wk; failed conservative therapy 2 to 4 wk	An Oswestry Low Back Pain Disability Questionnaire was administered before treatment and at intervals, the last at 6-mon follow-up. Patients and clinicians were blinded as to which treatment was performed.
Gautam et al.^30^	43	Acute radicular pain; failure of conservative therapies > 3 mon; evidence of contained disc herniation	Primary outcome measures included a VAS for pain and the Oswestry Disability Index. Secondary outcome measures included pain relief, reduction of analgesic consumption, and patient's satisfaction. Clinical assessment of these outcome measures was performed at 2 wk, 1, 3, 6 mon, and 1 yr after the procedure.
Das et al.^31^	40	Monoradicular pain; protrusion; contained disc; signs of disc degeneration; Oswestry Disability Index > 30%; failure to conservative therapies for 2 to 4 wk	Therapeutic outcome was assessed after 3 wk, 3, 6 mon, 1 and 2 yr on a VAS and Oswestry Low Back Pain Disability Questionnaire.
Paoloni et al.^32^	36	LBP and/or radiating pain of moderate to severe intensity (VAS > 5/10) to one leg; disc protrusion with or without disc degeneration in the spinal segments involved in the pain	Sixty patients suffering from acute LBP caused by LDH was randomized to an intramuscular O2O3 or control group. Patients were observed up to assess pain intensity, LBP-related disability, and drug intake (15 and 30 d after treatment started, and 2 wk, and 3 and 6 mon after treatment ended).
Perri et al.^33^	77	Lumbar disc protrusion ± herniation	During the following 6 mon, an MRI follow-up with the same sequences was performed. An IDVA, DWI signal score and post treatment clinical outcome evaluation were performed for an assessment of hernia reduction.
Rahimzadeh et al.^23^	20	American Anesthesiology Association I to II; age 20 -70 yr; at least 8 wk of LBP; pressure on the spinal cord neural roots; radiological confirmation; failed conservative therapy for 4 to 6	Patients were followed up for 12 mon regarding score on VAS and life performance improvement based on Oswestry Disability Index and satisfaction level.
Wu et al.^34^	108	Age 20-70 yr; LBP with leg pain; radiological confirmation of single level non-contained herniation; failure of conservative therapies in the prior 6 mon	Minimally invasive group of patients was treated with the injection of oxygen-ozone combined with collagenase into the lumbar disc or the epidural space; the other group was treated with traditional surgery. After the treatment, the patients were followed-up and the therapeutic effect was assessed at 2 wk, 3 and 12 mon by the modified Macnab criteria.
Zhang et al.^24^	90	LBP and radicular pain; single-level herniation; radiological confirmation; pain last at least 6 wk; failure of conservative therapies > 3 mon	VAS and JOA scores were administered before treatment and at 3-wk, 6- and 12- mon follow-up period to evaluate the clinical results.

DWI: Diffusion weighted imaging; IDVA: intervertebral disc volumetric analysis; JOA: Japanese Orthopedic Association; LBP: low back pain; MRI: magnetic resonance imaging; VAS: Visual Analogue Scale.

O_3_ therapy demonstrates considerable promise as a minimally invasive modality for IVDD, particularly in addressing refractory discogenic pain through its dual mechanisms of biochemical decompression and anti-inflammatory modulation. Building upon this foundation, emerging combinatorial strategies integrate O_3_ with advanced biomaterial platforms to enhance therapeutic precision. Notable innovations include O_3_-loaded nanoparticle delivery systems for sustained intradiscal release, temperature-response hydrogels co-administered with O_3_ to prolong tissue retention, and photothermally activated O_3_ generators that achieve spatiotemporally controlled oxidative effects. Such synergistic approaches aim to overcome limitations of monotherapy while amplifying regenerative outcomes. Critical to clinical translation is establishing standardized treatment protocols. This necessitates defining optimal O_3_ concentration gradients tailored to degeneration stages, implementing real-time imaging guidance for precise needle placement, and developing evidence-based dosing algorithms correlated with patient-specific factors including disc volume and hydration status. Multidisciplinary consensus on these parameters will determine the transition from experimental intervention to mainstream therapeutic option.

### Hyperbaric oxygen

HBO therapy, a widely employed clinical modality, is well-established to promote angiogenesis and enhance tissue oxygenation. HBO therapy is a medical intervention involving the inhalation of pure oxygen at pressures exceeding 1 atmosphere absolute, wherein artificially elevated ambient pressure enables the delivery of oxygen at partial pressures several-fold to orders of magnitude higher than normbaric conditions. For effective HBO, the oxygen pressure must be maintained at or above 1.4 atmosphere absolute (ATA). In monoplace chambers, the patient breathes 100% oxygen directly pressurized within the chamber.^35^ Evidence suggests that HBO alleviates pain in patients with lumbar disc herniation.^36^,^37^ Perioperative HBO effectively mitigates residual postoperative symptoms and reduces the incidence of nerve root edema, demonstrating beneficial effects in the treatment of lumbar disc herniation.^38^ Additional study showed combined HBO and lumbar paravertebral nerve block demonstrates significantly superior efficacy over nerve block alone in alleviating clinical pain symptoms and improving distal sciatic nerve conduction function in patients with lumbar disc herniation.^39^

A preclinical study demonstrated that HBO upregulated miR-107 expression in degenerated NP cells, thereby inhibiting Wnt3a/β-catenin signaling pathways.^40^ The cellular effects of HBO exhibit dose-dependent characteristics. This mechanism may confer protection against IVDD, suggesting HBO’s as a potential therapeutic intervention.^40^ The molecular composition and ultrastructural organization of the IVD extracellular matrix (ECM) govern its essential biomechanical properties. HBO treatment significantly upregulated ECM-related gene expression in NP cells, concurrently suppressing p38 mitogen-activated protein kinase phosphorylation while reducing catabolic or inflammatory factor production and elevating anti-catabolic factor levels.^41^ Studies demonstrated that HBO treatment significantly inhibited the production of IL-1β, prostaglandin E2, and NO while enhancing human and rat NP cell proliferation and ECM synthesis.^10^,^42^ Besides, HBO treatment inhibits mitogen-activated protein kinase signaling activation and suppresses the mitochondrial apoptotic pathway in degenerated human IVD cells.^43^ In human degenerative IVDs, HBO treatment of degenerated NP cells exerts a protective effect by mitigating apoptosis and its activation. The mechanism involved miR-573-mediated regulation of proliferation and apoptosis post-HBO treatment through direct targeting of Bax.^44^ These mechanistic benefits substantiate its emerging clinical application in IVDD, warranting further preclinical validation.

While HBO therapy has achieved clinical maturity in systemic conditions like carbon monoxide poisoning and wound healing, its application to focal avascular tissues such as IVD faces significant translational challenges. Precise spatial control of oxygen concentration and exposure kinetics within degenerate discs remains unresolved due to diffusion barriers inherent to cartilaginous endplates. Addressing this requires developing advanced delivery platforms, including targeted gas-carrying microbubbles or implantable oxygen-releasing hydrogels capable of sustaining localized hyperoxia microenvironments. Furthermore, optimizing combinatorial protocols with biologics or physical modalities necessitate rigorous determination of critical parameters—particularly pressure-time integrals and therapeutic windows relative to degeneration stages. Concurrently, establishing evidence-based clinical guidelines demands multicenter validation of standardized outcome metrics, including quantitative diffusion-weighted magnetic resonance imaging parameters and disc-specific biomarker profiles, to replace current empirical dosing practices.

## Therapeutic Gases in Preclinical Research of Intervertebral Disc Degeneration

### Hydrogen

H_2_ therapy is a promising antioxidation and anti-inflammatory approach. In 2007, a landmark study by Professor Shigeo Ohta (Nippon Medical School) in *Nature Medicine* demonstrated the neuroprotective effects of H_2_ inhalation in rodent stroke models, establishing the foundation for molecular H_2_ therapeutics.^45^ As the smallest diatomic molecule with high diffusion coefficient and nonpolar nature, H_2_ rapidly penetrates biological barriers, including plasma membranes and the blood-brain barrier, achieving rapid tissue distribution. This unique biodistribution profile enables selective antioxidant activity, mitochondrial modulation, and anti-inflammatory signaling, conferring therapeutic potential particularly for neurological disorders, ischemia-reperfusion injuries, and chronic pain syndromes.^46^ The therapeutic efficacy of many pharmaceutical agents is constrained by their large molecular mass, which impedes penetration through the blood-brain barrier and plasma membrane lipid bilayers, resulting in suboptimal bioavailability within encapsulated tissues like IVD. In contrast, molecular H_2_ exhibits unrestricted biodistribution owing to its ultralow molecular weight and high diffusion coefficient. As a highly selective antioxidant, H_2_ specifically scavenges cytotoxic radicals while preserving physiological ROS–a discriminatory capacity unattainable by conventional antioxidants such as vitamins C. This molecular selectivity ensures long-term administration does not disrupt redox homeostasis and confers superior matrix-protective effects in IVDD models.

**Figure 2:**
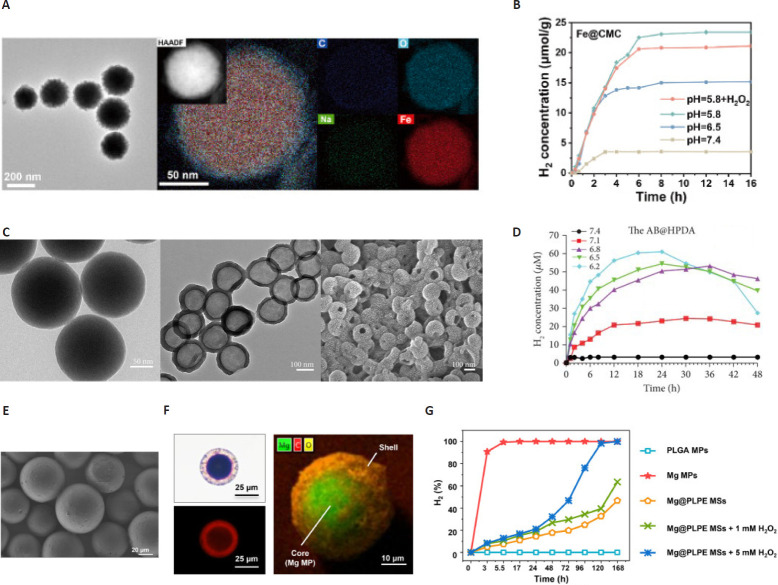
Characterization of H_2_-release materials. (A) SEM and EDX images of Fe@CMC. (B) Oscillatory strain sweep results of hydrogels. Reprinted from Ji et al.^48^ Copyright 2024 Elsevier Ltd. (C) Characterization of nanoparticles. (D) The cumulative release kinetics of H_2_ from the nanospheres in PBS (0.01 M, 37 °C) at various pH for 24 hours. Reprinted from Wang et al.^49^ (E) SEM image of Mg@PLPE MSs. (F) Fluorescence images of Mg@PLPE MSs with Nile red labeling. (G) Profiles of H_2_ generated from Mg@PLPE MSs in PBS with and without H_2_O_2_. Reprinted from Zhang et al.^50^ Copyright 2023 Acta Materialia Inc. AB@HPDA: Ammonia borane-loaded hollow polydopamine; C: carbon; EDX: energy-dispersive X-ray; Fe: iron; Fe@CMC: ferrous nanoparticles; H_2_: hydrogen; H_2_O_2_: hydrogen peroxide; HAADF: high-angle annular dark-field; Mg: magnesium; Mg@PLPE MSs: reactive oxygen species-responsive magnesium-containing microspheres; MP: microsphere; Na: sodium; O: oxygen; PBS: phosphate-buffered saline; SEM: scanning electron microscope.

H_2_ therapy has recently emerged as a promising therapeutic candidate, owing to its potent efficacy and favorable biosafety profile. H_2_ readily penetrates cellular membranes and accumulates in mitochondria, where it directly scavenges hydroxyl radicals (·OH) to eliminate ROS.^45^,^47^ H_2_ alleviates IVDD by restoring mitochondrial homeostasis: it suppresses excessive mitochondrial death responses (mitochondrial unfolded protein response and unselective mitophagy) while enhancing 5’-monophosphate-activated protein kinase-mediated mitochondrial biogenesis and controlled mitophagy^48^ (**Figure 2A** and **B**). An intelligent H_2_ nanogenerator was engineered using ferrous nanoparticles (Fe@CMC), elucidating the mechanistic basis of H_2_-mediated mitochondrial quality control coordination. This platform was synthesized via crosslinking between dopamine-functionalized oxidized konjac glucomannan and phenylboronic acid-conjugated hyaluronic acid, exhibiting dual pH/ROS responsiveness. The degenerative IVD microenvironment provides pathological activation stimuli through characteristic acidosis (pH ~6.5) and elevated ROS concentrations. H_2_ production occurred through the reaction of zero-valent iron with H ions, enabling prolonged H_2_ release from the nanogenerator (Fe@HP-OD) system while concurrently neutralizing local acidosis. H_2_ rebalances mitochondrial quality control by suppressing overactivated catabolic processes including the unfolded protein response and unselective mitophagy, while concurrently activating the adenosine 5’-monophosphate-activated protein kinase signaling pathway. This dual modulation coordinates mitochondrial biogenesis with controlled mitophagy to establish critical synthesis-degradation equilibrium.^48^

Another study on IVDD demonstrated that ammonia borane-loaded hollow polydopamine enables long-term controlled H_2_ release within the IVD, responsive to degeneration severity and pH. This effectively treats the condition by rebalancing oxidative stress and inflammation in the degenerative tissue^49^ (**Figure 2C** and **D**). To achieve endogenous stimulus-controlled H_2_ delivery within degenerated IVD, we engineered a pH-responsive ammonia borane nanoplatform. This precursor compound exhibits high H_2_ storage capacity (10 wt.%) and undergoes acid-catalyzed decomposition to release H_2_, thereby achieving spatiotemporally targeted anti-inflammatory effects through localized gas generation.

Conventional H_2_ delivery approaches, such as inhaled H_2_ gas, oral consumption of H_2_-saturated water, or intravenous infusion of H_2_-rich saline, demonstrate efficacy in treating gastrointestinal inflammation, malignancies, pulmonary disorders, and dermatological conditions. However, their therapeutic utility for IVDD is intrinsically limited by three pathoanatomic constraints: the low aqueous solubility of H_2_, the avascular microenvironment of adult discs, and the deep-seated pathology localized particularly within the central NP. Consequently, developing localized H_2_-generating systems capable of sustained release represents an imperative strategy to achieve therapeutic bioavailability in IVDD management. Therefore, another study designed a destruction of the hydrophobic shell enables aqueous penetration, triggering H_2_ generation through the magnesium-water reaction. This sustained H2 release actively scavenges cytotoxic ·OH radicals, effectively suppressing ROS accumulation. Notably, magnesium-containing microsphere attenuated H_2_O_2_/lipopolysaccharide-induced ROS overproduction, inflammatory responses, and H_2_O_2_-mediated ECM degradation in IVDD cells *in vitro*^50^ (**Figure 2E–G**). H_2_ release is initiated by the overproduced ROS (specifically H_2_O_2_) in the degenerated IVD microenvironment. This triggers a hydrophobic-to-hydrophilic transition in the ROS-responsive poly(lactic-co-glycolic acid) (PBT-co-EGDM) component of a ROS-responsive magnesium-containing microsphere (Mg@PLPE MS) shell, disrupting its integrity. Consequently, water penetrates the shell and contacts the magnesium core, initiating the hydrolysis reaction. The continuously generated H_2_ exerts its therapeutic effects primarily by selectively scavenging deleterious ROS, particularly ·OH. This effectively reduces oxidative stress, suppresses downstream inflammatory cascades, inhibits ECM degradation, and protects disc cells from apoptosis, thereby ameliorating IVDD, as validated both *in vitro* and *in vivo*.

### Hydrogen sulfide

H_2_S is a colorless, water-soluble gas characterized by a distinct odor of rotten eggs.^51^ It represents the third identified endogenous gaseous signaling molecule, following carbon monoxide and NO. Endogenous H_2_S is primarily generated by the cystathionine β-synthase and cystathionine γ-lyase.^52^,^53^ Additionally, it can be produced independently via the 3-mercaptopyruvate sulfurtransferase pathway, catalyzed by cysteine aminotransferase in the presence of α-ketoglutarate.^54^ H_2_S is synthesized endogenously in mammalian tissues and diffuses freely across cell membranes to exert pleiotropic biological effects in various systems. Substantial research has documented the therapeutic efficacy of H_2_S in atherosclerosis, cardiac remodeling, and myocardial ischemia or reperfusion injury. The underlying mechanisms encompass antioxidant effects, inhibition of apoptosis, promotion of angiogenesis, anti-inflammatory actions, and modulation of ion channels.^55^

**Figure 3:**
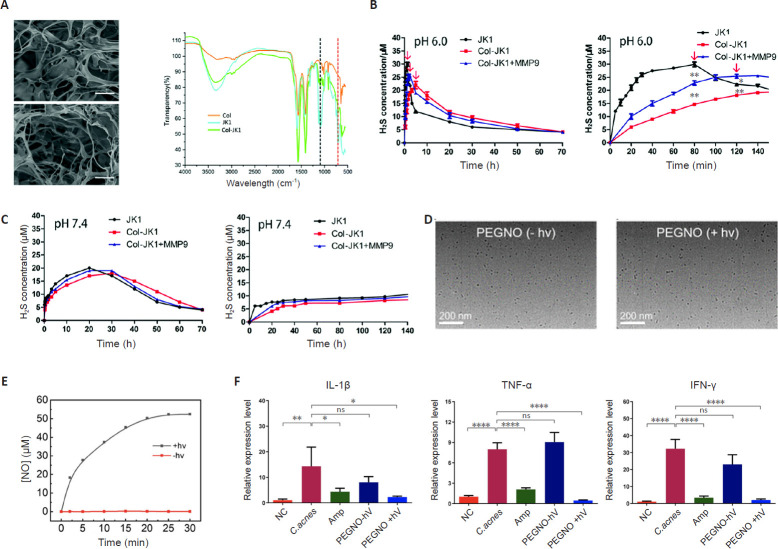
Characterization of H_2_S- and NO-release materials. (A) SEM images of collagen hydrogels without JK1 (Col) and with JK1 (Col-JK1), FTIR spectra of Col, JK1 and Col-JK1. (B, C) H_2_S release kinetics of JK1 and Col-JK1 under various conditions. Reprinted from Zheng et al.^65^ Copyright 2019 Royal Society of Chemistry. (D) TEM images of micelles before and after 630 nm irradiation for 30 minutes. (E) NO release profiles. (F) The expression of IL-1β, TNF-α, and IFN-γ from stimulated primary NP cells. Reprinted from Tao et al.^78^ Copyright 2022 American Chemical Society. *C. acnes*: *Cutibacterium acnes*; Col: collagen; Col-JK1: collagen-JK1 hydrogel; FTIR: Fourier transform infrared spectroscopy; H_2_S: hydrogen sulfide; IFN-γ: interferon-γ; IL-1β: interleukin-1β; JK1: a pH-responsive hydrogen sulfide donor; MMP9: matrix metallopeptidase 9; NC: normal control; NO: nitric oxide; PEGNO: polyethylene glycol-functionalized nitric oxide donor; SEM: scanning electron microscope; TNF-α: tumor necrosis factor-α.

H_2_S, an endogenous signaling molecule, exerts protective effects during IVDD. Exogenous supplementation of H_2_S donors can mitigate IVDD progression by mediating the antioxidant and anti-inflammatory properties of their organic and inorganic sulfide metabolites. The studies highlight H_2_S as a promising therapeutic agent for diverse pathologies, including cardiomyocyte injury, cutaneous wounds, and neurodegenerative disorders, owing to its critical roles in physiological and pathological processes.^56^,^57^,^58^,^59^,^60^ As an endogenously produced cytoprotective gas transmitter, H_2_S activates the phosphatidyqinositol-3 kinase/protein kinase B pathway in multiple injury models, thereby conferring cellular protection.^61^,^62^ Functioning as a gas transmitter and signaling molecule, H_2_S mediates cytoprotection against apoptosis through dual mechanisms: direct neutralization of cytotoxic reactive species (e.g., ROS and peroxynitrite) and enhanced expression of endogenous antioxidants (including superoxide dismutase, glutathione, and N-acetylcysteine).^63^ Sodium hydrosulfide, a widely employed H_2_S donor that rapidly elevates systemic H_2_S levels, was administered intravenously for in situ generation. However, its spontaneous hydrolysis in aqueous environments complicates precise dosing control. Furthermore, therapeutic efficacy is substantially restricted by rapid donor clearance from target sites and H_2_S volatilization, leading to unpredictable concentration decay in physiological solutions.^64^ To overcome these limitations, Zheng et al.^65^ developed a novel class of pH responsive H_2_S donors (phosphonamidothioates). Specifically, phosphonamidothioates 1 (JK1) liberates H_2_S selectively in acidic microenvironments (pH 5–6) through pH-triggered intramolecular cyclization, while remaining stable at physiological pH (7.4). Demonstrated efficacy in rat myocardial ischemia-reperfusion injury models confirms its therapeutic potential through targeted H_2_S release in acidic lesions. Given the analogous pathological acidification in IVDD, driven by glycolytic lactate accumulation, JK1 emerges as a promising candidate for IVDD treatment (**Figure 3A–C**). Both free JK1 and collagen-JK1 hydrogel (Col-JK1) exhibited pH-dependent H_2_S release kinetics. Acidic conditions accelerated H_2_S liberation, resulting in higher peak concentrations, earlier time-to-peak, and enhanced release rates. However, developing effective H_2_S prodrugs and achieving site-specific delivery to degenerated discs remain major challenges for future clinical translation.

**Figure 4:**
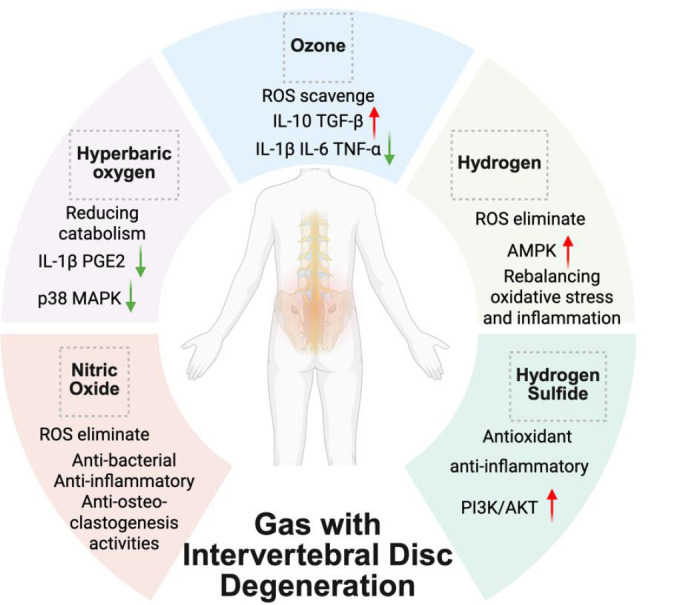
The mechanism of gas therapy in the application for intervertebral disc degeneration. Created with BioRender.com. AMPK: Adenosine monophosphate-activated protein kinase; IL: interleukin; p38 MAPK: p38 mitogen-activated protein kinase; PGE2: prostaglandin E2; PI3K/AKT: phosphoinositide 3’-kinase/protein kinase B; ROS: reactive oxygen species; TGF-β: transforming growth factor-β; TNF-α: transforming growth factor-α.

### Nitric oxide

NO, an endogenous gaseous mediator, plays a pivotal role in vasodilation, anti-angiogenesis, and immunomodulation. NO enhances the efficacy of chemotherapy for malignancies and ROS-dependent therapies, such as photodynamic therapy and chemical dynamic therapy. This enhancement occurs through the reaction of NO with ROS, generating reactive nitrogen species with potentiated oxidative and capabilities.^66^ NO functions as a critical intracellular messenger molecule involved in the pathogenesis of various conditions, such as infections,^67^ cerebrovascular diseases,^68^ and cirrhosis.^69^ NO supplementation during resuscitation following hemorrhagic shock enhanced microcirculatory perfusion in specific gastrointestinal regions early after treatment.^70^

Recent years have witnessed growing research interest in gas therapy utilizing endogenous signaling molecules such as NO for disease treatment. Beyond its physiological regulatory functions, NO mediates critical immunomodulatory roles in pathological contexts, including immune response regulation and wound healing promotion.^71^,^72^ Notably, NO serves as a broad-spectrum antimicrobial agent that circumvents drug resistance through distinct bactericidal mechanisms: induction of lipid peroxidation, DNA cleavage, and protein dysfunction.^73^ Additionally, NO reacts with superoxide radicals to generate reactive byproducts, which oxidatively disrupts bacterial membrane integrity, ultimately triggering lytic cell death. To achieve localized NO delivery, numerous stimuli-responsive NO donors have been engineered to mitigate systemic toxicity while enhancing therapeutic efficacy.^74^,^75^,^76^ Among these, photoresponsive NO-releasing molecules represent particularly promising candidates, attributed to the unparalleled spatiotemporal precision of light stimulation that enables exquisite controllability.^77^ A study demonstrates the successful fabrication of NO-releasing micellar nanoparticles via self-assembly of amphiphilic deblock copolymers bearing both palladium (II) meso-tetraphenylte trabenzoporphyrin photocatalyst and coumarin-derived NO donors within hydrophobic segments. This study also demonstrates that localized NO delivery mediates potent antibacterial, anti-inflammatory, and anti-osteoclastogenesis activities, thereby surpassing conventional antibiotics in treating IVDD with Modic changes in rat models (**Figure 3D–F**).^78^ Light-mediated activation represents a promising strategy for localized NO delivery. N-Nitrosoamine derivatives function as photoactive NO-releasing molecules that undergo photodissociation upon illumination, liberating NO. A photo redox catalytic approach was developed to trigger NO release within the phototherapeutic window (600–950 nm). This system operates under hypoxic conditions and enables red-light activation of ultraviolet-absorbing photo-activated NO releasing moieties (photoNORMs) through photocatalysis. While NO delivery systems are clinically established in cardiovascular and other fields, their exploration for IVDD remains notably scarce. NO exhibits well-characterized pleiotropic biological activities, including potent vasodilation, anti-inflammatory actions, modulation of ECM metabolism, and suppression of apoptosis.^79^,^80^,^81^,^82^ These properties confer significant therapeutic potential for mitigating key IVDD pathological processes such as impaired nutrient supply, chronic inflammation, matrix degradation, and disc cell death. However, realizing this potential necessitates the development of advanced delivery platforms. The inherent instability and paradoxical concentration-dependent effects of NO demand precision delivery strategies. Tailored biomaterial carriers or stimuli-responsive NO donors capable of providing sustained, localized, and controllable NO release within the harsh, avascular, and hypoxic microenvironment of the degenerated disc are, therefore, critical prerequisites for translating NO therapy into a viable clinical intervention for IVDD.

## Conclusion and Prospection

IVDD, a chronic progressive pathological process, presents a major clinical challenge. Degeneration-induced disc height reduction disrupts biomechanical homeostasis in adjacent spinal segments, culminating in secondary pathologies including spontaneous cervical spondylosis, chronic low back pain, sciatica, disc herniation, spinal stenosis, and myelopathy. These sequelae substantially compromise patients’ quality of life and work capacity, establishing IVDD as a leading cause of workforce attrition among adults. The precise etiopathogenesis of IVDD remains incompletely defined. However, current evidence supports a multifactorial interplay involving genetic predisposition, impaired nutrient supply, aberrant biomechanical loading, and dysregulated molecular signaling pathways.^83^ Current therapeutic approaches for IVDD encompass pharmacological management, physical modalities, and surgical interventions. Nevertheless, their clinical efficacy remains suboptimal, with a significant proportion of patients experiencing inadequate symptom relief and persistent functional impairment. This therapeutic inadequacy shows the imperative to elucidate the pathogenic cascades underlying IVDD progression, particularly dysregulated ECM metabolism, inflammatory microenvironment derangement and stress irregularity. Unraveling these mechanisms is critical for identifying novel therapeutic targets, thereby facilitating the development of precision strategies such as gene therapy, stem cell transplantation, and bioactive scaffolds—advances poised to fundamentally improve long-term functional outcomes. The emergence of gas therapy has introduced a novel therapeutic paradigm for IVDD. Bioactive gases, including H_2_, H_2_S and O_3_, leverage their unique pathophysiological modulatory capacities to reestablish disc microenvironmental homeostasis through mechanisms such as redox equilibrium regulation, inflammation suppression, and cell death, offering distinct mechanism-based advantages over conventional approaches.

Gas therapy has demonstrated considerable clinical utility in managing IVDD, with established applications including medical O_3_ for intradiscal disinfection, HBO for postoperative recovery, and O_2_-O_3_ nucleolysis for minimally invasive intervention. These approaches exploit the inherent bioactivities of therapeutic gases, such as anti-inflammatory effects, oxidative stress modulation, and enhanced tissue oxygenation, to alleviate discogenic pain and retard degenerative progression. To optimize clinical outcomes, future efforts must focus on standardizing administration parameters, developing evidence-based treatment protocols, and innovating combination strategies (**Table 2**) that integrate gas therapy with regenerative biomaterials or targeted drug delivery systems for synergistic efficacy. Gas therapy for IVDD demonstrates core therapeutic characteristics centered on oxidative stress modulation and inflammation resolution. It directly scavenges ROS and restores redox equilibrium, to mitigate oxidative damage. Concurrently, it suppresses aberrant inflammatory cascades through downregulation of key cytokines via NO signaling, while inhibiting inflammasome hyperactivity.^84^,^85^ Furthermore, these gases rehabilitate the disc microenvironment by enhancing nutrient diffusion and preserving ECM integrity via H_2_S-regulated collagen synthesis.^65^ These synergistic mechanisms, spanning molecular regulation to biomechanical restoration, collectively address IVDD multifactorial pathology with superior spatial specificity compared to conventional therapies.

**Table 2:** Comparative analysis of gas-based technologies for IVDD

Technology	Delivery method	Clinical stage	Key advantages	Major limitations
O_3_-nucleolysis	CT-guided IVD injectior	Widely applied in clinical practice	Widely applied in clinical practice	Concentration-dependent neurotoxicity risk
HBO	Gas	Widely applied in clinical practice	Increase blood oxygen content	The indications and oxygen concentration are not definite
H_2_-loaded hydrogel	ROS scavenging/mitochondrial protection	Phase II trials	Sustained release (> 14 d)/ECM regeneration promotion	Limited penetration depth (< 5 mm)
H_2_S-nanogenerator	Caspase-3 inhibition/Anti-apoptotic	Preclinical validation	Disease site-specific activation/disc-targeting efficiency	Long-term biosafety pending verification
NO-MSC	Mitophagy induction/immunomodulation	Preclinical validation	Synergistic regenerative remodeling/microenvironment resetting	Complex manufacturing

CT: Computed tomography; ECM: extracellular matrix; H_2_: hydrogen; H_2_S: hydrogen sulfide; HBO: hyperbaric oxygen; IVD: intervertebral disc; IVDD: intervertebral disc degeneration; NO-MSC: nitric oxide-mesenchymal stem cells; O_3_: ozone; ROS: reactive oxygen species.

However, gas treatment on IVDD preclinical research remains constrained by significant limitations: current investigations are predominantly restricted to H_2_, H_2_S, and NO. Moreover, studies rely heavily on rodent models that inadequately recapitulate human disc pathophysiology. Advancing this field necessitates deepened mechanistic exploration of gas-specific molecular pathways, such as H_2_-mediated nuclear factor erythroid 2-related factor 2 antioxidant activation, H_2_S dependent ECM regulation, anti-apoptotic signaling within human disc cell microenvironments, coupled with multidimensional validation using physiologically relevant models, particularly human disc organoids and large animal models under biomechanical loading.^86^ Critical translational challenges include engineering NP-targeted delivery platforms responsive to degenerative niches, such as hypoxia-activated gas donors or ROS-triggered H_2_S nanogenerators. The strategic convergence of gas biology and precision engineering represents a paradigm-shifting therapeutic modality for IVDD, offering potential to overcome limitations of conventional pharmacologic and surgical interventions through this multidisciplinary approach. Sulfur dioxide (SO_2_), transitioning from pollutant to endogenous gas transmitter, enables tumor-specific oxidative lethality via glutathione depletion and ROS amplification. Its deep tissue penetrance and responsive delivery systems facilitate precise apoptosis induction through p53/Bax upregulation and Bcl-2 suppression.^87^ Complementarily, carbon monoxide exerts dual antitumor actions: mitochondrial dysfunction-mediated apoptosis and the tumor-immune microenvironment remodeling via immunomodulation.^88^ Collectively, unexplored therapeutic gases hold significant potential for preclinical IVDD research.

Advances in micro-nano engineered biomaterial carriers have enabled targeted molecular delivery through enhanced molecular loading capacity stimulus-responsive behavior and site-specific transport.^89^,^90^ These platforms are extensively utilized in nanomedicine and pharmaceutical development. Such technological progress provides foundational support for precision gas therapy which involves loading gaseous molecules or their precursors onto bio-carriers to construct stimulus-triggered release systems for disease-targeted treatment.^91^ Integrating gas-responsive release mechanisms with medical imaging guidance and targeted delivery pathways offers a promising paradigm for achieving therapeutic interventions with high efficacy and favorable safety profiles.^92^ Therefore, the development of gaseous agent-functionalized material hybrids and their combined application is poised to become a major research focus and a novel therapeutic strategy. This integrated approach holds significant promise for overcoming the limitations of single-modality therapies and enabling more precise and effective targeted treatments.

From the perspective of precision medicine, integrating multifunctional carriers with gaseous agents or gas prodrugs to develop stimuli-responsive intelligent gas-drug systems holds significant promise for achieving precise gas therapy of diseases. The advancement of multifunctional carriers provides a technological foundation for targeted gas delivery and controllable release. The paramount advantage of such stimuli-responsive systems lies in their ability to achieve spatiotemporally controlled drug release through exogenous or endogenous triggers, such as external stimuli (e.g., light, ultrasound, electricity, and magnetism) or internal stimuli (e.g., redox conditions, pH gradients, and specific enzyme levels). This site-specific release minimizes systemic drug exposure, thereby substantially reducing adverse effects.^93^,^94^,^95^ Further research will focus on exploiting these dual exogenous-endogenous responsive mechanisms for therapeutic applications. Clinical translation of gas therapy is highly feasible given favorable metabolic pathways and established biosafety profiles, exemplified by medical O_3_’s rapid conversion to oxygen within disc tissue. O_3_ therapy demonstrates excellent clinical safety in intradiscal applications for disinfection and anti-inflammation, with minimal systemic toxicity when administered at therapeutic concentrations. For emerging gases, stimuli-responsive delivery platforms further enhance biosafety by confining release to degenerative niches while maintaining therapeutic efficacy. This study focused on established gases with limited coverage of emerging mediators. Clinical evidence relies heavily on small-scale studies, lacking robust randomized controlled trial validation. Species differences between rodent models and human IVDD constrain mechanistic translation. Delivery precision challenges and long-term biosafety data for novel gas-releasing biomaterials require further investigation. Standardized therapeutic protocols remain underdeveloped. Additionally, the references search was restricted to PubMed and English-language publications, which may introduce selection bias and incomplete retrieval.

Collectively, gaseous molecule-based therapies show promising potential for IVDD management, though current evidence remains exploratory. Future research should prioritize: (1) large-scale multicenter randomized controlled trials to rigorously validate efficacy and safety profiles; (2) mechanistic investigations elucidating therapeutic actions; (3) subtype-specific efficacy analyses across IVDD etiologies to enable personalized regimens; (4) combinatorial strategies with conventional therapies for synergistic outcomes; and (5) advanced delivery platforms (e.g., functionalized nanomaterials) to enhance therapeutic precision. These initiatives will accelerate clinical translation of gas therapy for IVDD. This review comprehensively delineates gas therapeutics (O_3_, HBO, H_2_, H_2_S, and NO) for IVDD, integrating clinical applications with preclinical mechanistic insights (**Table 3**).^43^,^44^,^49^,^50^,^65^,^78^,^96^,^97^,^98^ Core advantages include minimally invasive targeted delivery and multimodal bioregulation. Future research priorities include deciphering pathologically distinct microenvironmental targets; engineering stimuli-responsive nano delivery platforms; pioneering combinatorial regimens—gas-stem cell synergy, gas-scaffold composites, and spatiotemporal sequential gas release systems. Such multimodal integration will maximize the translational potential of gaseous therapeutics in precision IVDD management. Future combinatorial strategies should prioritize gas-stem cell synergy, such as O_3_-preconditioned mesenchymal stem cells to enhance antioxidant capacity and survival within hypoxic discs and gas-scaffold composites, such as H_2_S-releasing hydrogels that concurrently modulate inflammation and promote ECM regeneration. These integrated approaches leverage bidirectional crosstalk, gaseous mediators’ prime regenerative cells while functionalized scaffolds provide spatiotemporal control of bioactive gas release, synergistically restoring disc homeostasis through amplified anti-inflammatory, antioxidant, and anabolic actions—collectively bridging translational gaps in IVDD therapy.

**Table 3:** Gas therapeutics (O_3_, HBO, H_2_, H_2_S, and NO) for IVDD

Gas	Gas administration	Disease	Mechanism	Reference
O3	O_2_/steroid	Degenerative spinal disease	A functional and sustained analgesic effect	96
	Intradiscal O_3_ treatment	Low back pain	Anti-inflammatory properties	97
	Percutaneous O_3_ nucleolysis	Lumbar disc herniation	An oxidizing effect on the proteoglycans	98
HBO	Hyperbaric oxygen therapy	Modic changes	Antimicrobial bactericidal and/or bacteriostatic effects	43
	Combined with transfection with anti-miR-573	IVDD	Improve hypoxic conditions by increasing tissue and/or microvascular O_2_ levels	44
H_2_	A pH-responsive delivery of H_2_ through ammonia borane-loaded hollow polydopamine	IVDD	Inhibit IVDD by reducing oxidative stress and inflammation and promoting ECM secretion	49
	A ROS-responsive magnesium-containing microsphere	IVDD	Anti-inflammatory effects and alleviated apoptosis	50
h_2_s	A pH and enzyme dual-responsive H_2_S	IVDD	Anti-inflammatory effects through the regulation of the NF-kB signaling pathway	65
NO	Self-assembly of PEGNO block copolymers into NO-releasing micelles	IVDD	Antibacterial, anti-inflammatory, and anti-osteoclastogenesis effects	78

H_2_: Hydrogen; H_2_S: hydrogen sulfide; HBO: hyperbaric oxygen; IVDD: intervertebral disc degeneration; miR-573: microRNA 573; NF-kB: nuclear factor kB; NO: nitric oxide; O_2_: ozone; ROS: reactive oxygen species.

## Data Availability

*Not applicable*.
